# Deaths due to *Plasmodium knowlesi* malaria in Sabah, Malaysia: association with reporting as *Plasmodium malariae* and delayed parenteral artesunate

**DOI:** 10.1186/1475-2875-11-284

**Published:** 2012-08-20

**Authors:** Giri S Rajahram, Bridget E Barber, Timothy William, Jayaram Menon, Nicholas M Anstey, Tsin W Yeo

**Affiliations:** 1 Infectious Diseases Department, Queen Elizabeth Hospital, Karung Berkunci No. 2029, Jalan Penampang, Kota Kinabalu, 88560, Sabah, Malaysia; 2 Menzies School of Health Research and Charles Darwin University, Darwin, NT, Australia; 3 Department of Medicine, Queen Elizabeth Hospital, Kota Kinabalu, Sabah, Malaysia; 4 Sabah Department of Health, Kota Kinabalu, Sabah, Malaysia; 5 Royal Darwin Hospital, Darwin, NT, Australia

**Keywords:** Malaria, *Plasmodium knowlesi*

## Abstract

**Background:**

The simian parasite *Plasmodium knowlesi* is recognized as a common cause of severe and fatal human malaria in Sabah, Malaysia, but is morphologically indistinguishable from and still commonly reported as *Plasmodium malariae,* despite the paucity of this species in Sabah. Since December 2008 Sabah Department of Health has recommended intravenous artesunate and referral to a general hospital for all severe malaria cases of any species. This paper reviews all malaria deaths in Sabah subsequent to the introduction of these measures. Reporting of malaria deaths in Malaysia is mandatory.

**Methods:**

Details of reported malaria deaths during 2010-2011 were reviewed to determine the proportion of each *Plasmodium* species. Demographics, clinical presentations and management of severe malaria caused by each species were compared.

**Results:**

Fourteen malaria deaths were reported, comprising seven *Plasmodium falciparum*, six *P. knowlesi* and one *Plasmodium vivax* (all PCR-confirmed). Of the six *P. knowlesi* deaths, five were attributable to knowlesi malaria and one was attributable to *P. knowlesi*-associated enterobacter sepsis. Patients with directly attributable *P. knowlesi* deaths (N = 5) were older than those with *P. falciparum* (median age 51 [IQR 50-65] *vs* 22 [IQR 9-55] years, p = 0.06). Complications in fatal *P. knowlesi* included respiratory distress (N = 5, 100%), hypotension (N = 4, 80%), and renal failure (N = 4, 80%). All patients with *P. knowlesi* were reported as *P. malariae* by microscopy*.* Only two of five patients with severe knowlesi malaria on presentation received immediate parenteral anti-malarial treatment. The patient with *P. vivax*-associated severe illness did not receive parenteral treatment. In contrast six of seven patients with severe falciparum malaria received immediate parenteral treatment.

**Conclusion:**

*Plasmodium knowlesi* was responsible, either directly or through gram-negative bacteraemia, for almost half of malaria deaths in Sabah. Patients with severe non-falciparum malaria were less likely to receive immediate parenteral therapy. This highlights the need in Sabah for microscopically diagnosed *P. malariae* to be reported as *P. knowlesi* to improve recognition and management of this potentially fatal species. Clinicians need to be better informed of the potential for severe and fatal malaria from non-falciparum species, and the need to treat all severe malaria with immediate intravenous artesunate.

## Background

The simian parasite *Plasmodium knowlesi* is commonly misdiagnosed as *Plasmodium malariae* by microscopy due to its near-identical appearance. However, in contrast to the relatively benign clinical course of *P. malariae, P. knowlesi* is now recognized as a common cause of severe and fatal human malaria in Malaysian Borneo [1]. Cases have also been reported in West Malaysia [2] and nearly all countries in Southeast Asia [3-10]. In Borneo, *P. knowlesi* mono-infection accounted for 64% of malaria admissions in Kapit, Sarawak [11], and 78% of malaria admissions in Kudat, Sabah [12]. Severe disease has been reported from Sarawak [1,11], Sabah [13,14], and West Malaysia [2], including 13 fatal cases [1,11,13,14]. In a retrospective study conducted from December 2007 to November 2009 at Queen Elizabeth Hospital (QEH), a tertiary referral hospital in Sabah, 22/56 (39%) patients admitted with PCR-confirmed knowlesi malaria had severe disease by WHO criteria, and six (27%) died [14].

The 24-hour replication cycle of *P. knowlesi* may be associated with rapid increases in parasitaemia and consequent complications, and hence prompt diagnosis and initiation of effective treatment is essential. The optimal treatment however has not been determined. While chloroquine was shown to be effective for uncomplicated knowlesi malaria in Kapit [15], the retrospective study at QEH found faster parasite clearance times with an oral artemisinin combination therapy (ACT), artemether-lumefantrine [14]. Among patients with severe knowlesi malaria, parasite clearance times were faster with intravenous artesunate than with intravenous quinine, and fewer patients who received artesunate died [14].

In Sabah, intravenous artesunate has been the recommended treatment for severe malaria from any species since December 2008. In addition, referral to a general hospital is advised for patients with symptoms or signs suggestive of severe disease. Despite these measures, 14 deaths from malaria were reported in Sabah during 2010-2011. In this study the case notes of all these patients were reviewed to determine the *Plasmodium* species causing fatal malaria in Sabah, and to identify any notable differences in demographics, clinical features and management of fatal malaria caused by the different species.

## Methods

### Setting

The north-eastern Malaysian state of Sabah has an area of 73,600 km^2^ and a population of 3.2 million [16]. Situated between 4° and 7° above the equator, Sabah has a mostly tropical climate, with high humidity and rainfall throughout the year and temperatures of 25-35°C. Malaria incidence is estimated at 0.78/1000 persons/year [17]. Sabah’s government health system comprises one tertiary-referral hospital offering specialist and sub-specialist care, three general hospitals offering specialist care, 18 district hospitals, 77 health clinics and 189 rural clinics.

### Case series

All deaths due to malaria in Sabah must be reported to the Sabah Ministry of Health where they are reviewed. Details of reported malaria deaths during 2010-2011 were obtained from the Sabah Department of Health. Approval to review the case notes of fatal malaria cases was obtained from the Medical Research sub-Committee of the Malaysian Ministry of Health and the Health Research Ethics Committee of Menzies School of Health Research. Case notes were retrieved from district hospitals and reviewed for clinical details, laboratory results and cause of death.

Blood slides for malaria parasites were reported according to a scale of 1+ to 4+ (1 + = 1-10 parasites/100 high power microscopy fields [HPMFs] or ≈ 4-40 parasites/μL, 2 + = 11-100 parasites/100 HPMFs or ≈ 41-400 parasites/μL, 3 + = 1-10 parasites/HPMF or ≈ 401-4,000 parasites/μL, and 4+ = >10 parasites/HPMF or >4,000 parasites/μL). PCR was performed by the Sabah State Reference Laboratory by methods previously published [1,18]. The diagnosis of all fatal malaria cases was confirmed by PCR. Laboratory investigations and clinical details are listed in Table 1.

**Table 1:** **Demographic, clinical and laboratory features of reported *Plasmodium knowlesi *deaths, on admission **

**Details**	**Case 1**	**Case 2**	**Case 3**	**Case 4**	**Case 5**	***P. knowlesi*-associated gram-negative septic shock**
Age, years	71	65	51	50	49	36
Sex	Male	Male	Male	Male	Female	Female
Time to death, hours	1	75	16	56	63	84
Blood pressure, mmHg	75/49	117/73	70/40	83/51	112/51	131/87
Heart rate per minute	127	58	110	89	113	110
Oxygen saturation on room air, %	75	92	90	60	88	85
PaO2:FiO2 ratio	NA	NA	66	115	91	NA
Axillary temperature, °C	39.4	36.8	37.6	37.5	36.7	37.8
Haemoglobin, g/dL (females 12.0-16.0, males 13.5-17.5)	14.9	14.6	9.4	10.9	12.6	11.1
WBC count, x 10^3^ cells/μL (4.5-11)	10.8	4.8	6.8	7.81	12.1	7.8
Platelet count, x 10^3^ cells/μL (150-450)	88	58	8	3	32	53
Serum creatinine, μmol/L (63-133)	1451	NA	578	330	283	NA
Serum urea, mmol/L (1.0–8.3)	81.5	6.1	44	38.5	25	10.8
Total serum bilirubin, μmol/L (<17)	NA	NA	146	74	25	NA
Serum aspartate aminotransferase, U/L (<37)	42	NA	53	NA	39	NA
Serum alanine aminotransferase concentration, U/L (<40)	NA	NA	28	49	20	NA
Serum albumin, g/L (35-60)	NA	NA	23	19	28	NA
Serum bicarbonate, mmol/L (18-23)	NA	NA	14	16.2	14	6.5
Serum lactate mmol/L (0.5-2.2)	NA	NA	6.4	6.8	NA	NA
Blood cultures	negative	Not done	negative	negative	Not done	*Enterobacter cloacae*
Initial microscopic diagnosis	*P. falciparum”3+” P. malariae* “4+”	*P. malariae* “3+”	*P. malariae* “4+”	*P. vivax* “4+”	*P. malariae* “4+”	*P. malariae* “1+”
PCR result*	*P. knowlesi*	*P. knowlesi*	*P. knowlesi*	*P. knowlesi*	*P. knowlesi*	*P. knowlesi*
Initial therapy received	IV quinine/oral chloroquine, primaquine and doxycycline	Oral chloroquine	IV artesunate	Oral chloroquine and primaquine	Oral chloroquine and primaquine	Oral sulfadoxine/ pyrimethamine and primaquine

**NOTE.** Laboratory reference ranges are given in parentheses.

*NA*, not available; *IV*, intravenous.

*Blood samples used for PCR-confirmation were taken on the day of hospital admission for cases 1, 3 and 5; day 2 for case 2; day 3 for case 4, and day 1 for case 6.

## Results

Fourteen deaths were reported during 2010-2011, including five due to *P. knowlesi* mono-infection, one *P. knowlesi-*associated fatality in which gram-negative septic shock was thought to be the primary cause of death, one death associated with *P. vivax* and seven from *P. falciparum*.

### Cases 1-5: fatal cases primarily attributed to PCR-confirmed *plasmodium knowlesi* monoinfection

#### Case one

A 71-year-old man with a history of hypertension and chronic obstructive pulmonary disease presented to a district hospital with a seven-day history of fever and dyspnoea, and reduced conscious state just prior to presentation. His Glasgow Coma Score (GCS) was recorded on arrival by a paramedic as 3/15 and shortly afterwards as 10/15; his blood pressure was 75/49 mmHg and his oxygen saturation was 75% on room air. Chest auscultation noted prolonged expiratory phase but no crackles or wheeze. Blood film was reported as *Plasmodium falciparum/P. malariae* “3+”, and renal failure was present. Chest radiograph showed no infiltrates, and arterial blood gas was not performed. He was commenced on intravenous fluids and quinine in addition to oral primaquine, chloroquine and doxycycline. His oxygen saturation and blood pressure continued to deteriorate and he suffered a cardiac arrest 40 minutes after presentation. PCR confirmed *P. knowlesi* mono-infection.

#### Case two

A 65-year-old man with a history of type 2 diabetes and hypertension presented to a district hospital with a two-day history of fever, lethargy, myalgia and retro-orbital pain. On examination he was alert and orientated but hypoxic with an oxygen saturation of 92% on room air. Initial blood film was reported as negative for malaria parasites, and the patient was given a provisional diagnosis of dengue fever and commenced on intravenous fluids. On day 2 a repeat blood film was reported as *P. malariae* “3+” and oral chloroquine was started. The following day the patient was noted to be tachypnoeic and hypoxic (oxygen saturation 85% on room air), and chest radiograph showed diffuse infiltrates. Emergency intubation was performed however cardiac arrest occurred during the procedure. Cause of death was reported as severe malaria with acute respiratory distress syndrome.

#### Case three

A 51-year-old man with no known medical history presented to a clinic with a four-day history of fever, rigours, myalgia and arthralgia. On examination he was drowsy, jaundiced, hypotensive (blood pressure 70/40 mmHg), tachycardic (heart rate 110 beats/minute) and hypoxic (oxygen saturation 90% on room air). Hepatomegaly was noted on abdominal examination. Blood film was reported as *P. malariae* “4+”, and he had renal failure with a creatinine of 578 μmol/L. Chest radiograph revealed patchy consolidation in both lung fields. He was commenced on intravenous fluids, oxygen supplementation, inotropic support, intravenous antibiotics and intravenous artesunate, and transferred to a general hospital, where he was intubated and ventilated and commenced on haemodialysis. He died the following day from acute respiratory distress syndrome. Blood cultures were negative.

#### Case four

A 50-year-old man with no known medical history presented to a district hospital with a seven-day history of fever and rigours and a three-day history of cough. On examination he was alert and orientated but hypotensive (blood pressure 83/51 mmHg) and hypoxic (oxygen saturation 70% on 10 L oxygen via high flow mask), and wheeze was heard on chest auscultation. Blood film was reported as *Plasmodium vivax* “4+”. The patient was commenced on intravenous fluids, antibiotics and oral chloroquine, and transferred to a general hospital, where he was intubated and ventilated, and commenced on inotropic support. Chest radiograph showed diffuse infiltrates. The patient had renal failure (creatinine 330 μmol/L) and severe thrombocytopenia (3 x 10^3^ platelets/μL) although no bleeding complications were noted. Haemodialysis was commenced and platelet transfusion given. On day 3 a repeat blood film was reported as *P. malariae* “4+”. Intravenous artesunate was commenced, however the patient remained on maximum inotropic and ventilator support, and further haemodialysis was temporarily unavailable. He died nine hours later with multiple organ failure. Cause of death was reported as severe malaria. Blood cultures and dengue serology were negative, and PCR performed on a blood sample taken on day 3 confirmed *P. knowlesi* monoinfection.

#### Case five

A 49-year-old woman with no known medical illness presented to a district hospital with a four-day history of fever, cough and dyspnoea. On examination she was tachypnoeic and hypoxic (oxygen saturation on room air 88%) and wheeze was heard on chest auscultation. Blood film was reported as *P. malariae* “4+”, and renal failure was present (creatinine 283 μmol/L). Bronchodilators, intravenous antibiotics, oral chloroquine and primaquine were commenced in addition to inotropic support and transfer to a tertiary hospital. Chest radiograph on arrival revealed bilateral lower zone infiltrates, and the patient was intubated and ventilated however developed refractory hypotension and died the following morning. Cause of death was stated as septic shock, although blood cultures were not performed.

### Case 6: fatality attributed to *Plasmodium knowlesi*-associated gram-negative sepsis

A 36-year-old woman with no known medical illness presented to a district hospital with a seven-day history of fever, cough and myalgia. Examination was unremarkable and no features of severe malaria were evident. Blood film was reported as *P. malariae* “1+” and she was treated with oral sulphadoxine/pyrimethamine (SP) and primaquine. On day 3, she was aparasitaemic, but had become tachypnoeic (respiratory rate 44 breaths/minute), hypoxic (oxygen saturation 85% on room air) and hypotensive, and widespread wheeze was heard on chest auscultation. Chest radiograph showed no infiltrates and arterial blood gas revealed metabolic acidosis (PaO_2_ 110 mmHg, pH = 7.21, bicarbonate 6.5 mmol/L). She was commenced on bronchodilators, intravenous ceftriaxone and inotropic support and transferred to a tertiary hospital where she was intubated and ventilated and given intravenous artesunate. Further investigation results at the tertiary hospital included haemoglobin 8.0 g/dL, platelets 65 x 10^3^/μL, white cell count 6.6 x 10^3^/mL, creatinine 149 μmol/L and aspartate aminotransferase 795 U/L. Following admission the patient’s blood pressure deteriorated further and she developed a tachyarrthymia. Cardioversion was unsuccessful and the patient died from cardiac arrest four hours after arrival. Cause of death was reported as severe malaria, however on review, blood cultures taken on admission to the district hospital were noted to be positive for *Enterobacter cloacae* (reported as sensitive to ceftriaxone), untreated until progression to septic shock. Repeat cultures taken after antibiotics at the referral hospital were negative.

### Case 7: fatality associated with vivax malaria

An 85 year-old-woman presented to a district hospital with fever, rigours and abdominal pain. On examination she was pale, tachypnoeic and hypoxic with oxygen saturation of 88% on room air. Her blood pressure was 137/88 mmHg, and chest auscultation was clear. No arterial blood was available. On abdominal examination a tender pulsating mass was noted, and bedside ultrasound confirmed a 7.6 cm x 5.6 cm abdominal mass with minimal free fluid. No computed tomography scan was done. A provisional diagnosis of aortic dissection was made, and after discussion with family, conservative management was planned. Blood investigations revealed anaemia (haemoglobin 8.8 g/dL), thrombocytopenia (platelets 15 x 10^3^/μL) and acute kidney injury (creatinine 149 μmol/L, urea 28 mmol/L). Blood film was reported as *P. malariae* “4+”, and oral chloroquine was commenced. Her condition deteriorated with increasing oxygen requirements and on day 3 her blood pressure became unrecordable. The cause of death was reported as dissecting aortic aneurysm. PCR identified *P. vivax* mono-infection.

### Cases 8-14: fatal falciparum malaria

The falciparum deaths comprised three children (aged eight to 11 years) and four adults (aged 22–60 years). All the children and two adults were Filipino. All met the WHO criteria for severe malaria [19] on presentation, with severity criteria including jaundice (N = 5) renal failure (N = 4), respiratory distress (N = 4), anaemia (N = 4), hypotension (N = 3) and cerebral malaria (N = 1). One patient died without receiving anti-malarial treatment due to a delayed diagnosis, but all others were given intravenous quinine (N = 3) or artesunate (N = 3) within two hours of malaria diagnosis. Three patients were intubated and ventilated, three received inotropes and two were dialyzed. Four patients died within one day of presentation while three died at days 2-5. Blood cultures in one child were positive for *Enterobacter aerogenes.*

## Discussion

This case series highlights the misdiagnosis of severe knowlesi malaria due to continued reporting of *P. knowlesi* as *P. malariae*. It also highlights the lack of recognition of non-falciparum *Plasmodium* species as potential causes of severe and fatal malaria, and the fatal consequences of initial oral therapy for severe malaria of any species, particularly *P. knowlesi*.

In this series patients with deaths directly attributable to *P. knowlesi* were older than those with fatal *P. falciparum* (median age 51 [IQR 50-65] *vs* 22 [IQR 9-55] years, p = 0.06). The older age group affected, and the complications experienced by the fatal knowlesi malaria patients, are consistent with those already reported [1,11,14]. In particular, respiratory distress (oxygen saturation <94% and respiratory rate >30 breaths/minute) occurs commonly in knowlesi malaria [11,14], and was present in all patients. Diffuse infiltrates were seen on chest radiograph in four patients, and three of these patients met the criteria for acute respiratory distress syndrome (ratio of the partial pressure of oxygen to the fraction of inspired oxygen [PaO2:FiO2] <200). All patients required ventilatory support, and respiratory failure contributed directly to cause of death in at least four patients. Renal failure has been reported to occur in 30–55% of patients with severe knowlesi malaria [11,14], and occurred in four of five (80%) patients in this series.

Hypotension is also a common complication of severe knowlesi malaria, and occurred in four of five (80%) patients with deaths directly attributable to *P. knowlesi*. Pre-antibiotic blood cultures were taken in three of these patients and were all negative. Relatively low rates of clinically significant bacteraemia have been reported previously in severe knowlesi malaria [14], suggesting that bacteraemia is unlikely to account for hypotension in most patients with *P. knowlesi*. Nevertheless, one patient in this series who presented with uncomplicated knowlesi malaria had *Enterobacter cloacae* bacteraemia on admission, with bacterial septic shock the likely cause of death. This species was also identified in the only other report of clinically significant bacteraemia in a patient with severe knowlesi malaria [14]. Gram-negative bacteraemia is a well-recognized complication of severe falciparum malaria in children, and is associated with increased mortality [20-25]. The proposed mechanisms of the association between falciparum malaria and bacteraemia include an increased risk of non-typhoidal *Salmonella* due to malaria-induced haemolysis and neutrophil dysfunction [26], impaired macrophage function due to haemozoin deposition in monocytes [27-29], and nitric oxide quenching [30]. Bacterial translocation into the blood stream has also been hypothesized as a result of microvascular sequestration of *P. falciparum* in gut mucosa, and parasite accumulation in multiple organs was demonstrated in the single *P. knowlesi* autopsy report. Further studies are required to investigate mechanisms of bacteraemia in *P. knowlesi* infection.

Coma has not been reported to occur in knowlesi malaria, despite the only autopsy report demonstrating cerebral accumulation of parasitized red blood cells [13]. One patient in this study was reported to have had reduced consciousness and hypotension just prior to death. His altered conscious state however likely reflected his agonal state and is not consistent with cerebral malaria.

In contrast to other human malarias, *P. knowlesi* has a 24-hour replication cycle, which can lead to rapid increases in parasitaemia. Diagnosis of this malaria species is therefore critical in order that its potential to cause severe disease is recognized, appropriate treatment instituted without delay, and further complications and fatalities avoided. In this series, no patient was correctly diagnosed with *P. knowlesi*. Rather, all received a diagnosis of *P. malariae*, despite a very low incidence of this species in Sabah, with *P. malariae* detected by nested PCR in only 2 of 318 (0.6%) microscopy-diagnosed *P. malariae* cases referred to the Sabah State Public Health Laboratory in 2009 [31]. On thick film microscopy, *P. knowlesi* is indistinguishable from *P. malariae*. While there may be very subtle morphological differences between the late stages of *P. knowlesi* and *P. malariae* on thin film microscopy, these are not consistent and cannot be relied upon in clinical practice [32]. *P. malariae* causes a relatively benign acute illness, with low parasitaemia and only very rare reports of acutely severe malaria [33,34]. Current textbook descriptions and treatment guidelines reflect this. As has been previously recommended [1], this case series highlights the need for blood films positive for malaria parasites resembling *P. malariae* to be reported as *P. knowlesi* in Sabah, in order to direct clinicians in the recognition and management of this potentially fatal species.

Moreover, this case series illustrates the importance of a unified treatment policy for all patients with severe malaria, regardless of species [35]. Although the optimal treatment of knowlesi malaria has not been determined, severe disease should be treated as for severe falciparum malaria, including immediate institution of parenteral artesunate. *Plasmodium knowlesi* is also sometimes misdiagnosed as *P. vivax* (as occurred initially in one patient in this series) and *P. vivax* can also cause severe and fatal disease [36,37]. For both these reasons, *P. vivax* should also be treated with intravenous artesunate if signs of severity are present, particularly as oral chloroquine may be poorly absorbed in acutely unwell patients and chloroquine-resistant *P. vivax* is increasingly prevalent in Southeast Asia [38]. This unified treatment strategy for severe malaria from all species, including *P. knowlesi*, has been Sabah state policy since 2008 and has been adopted in the latest WHO severe malaria management guidelines [35]. Despite this policy, in this case series only two of five patients with knowlesi malaria who met severity criteria on admission received immediate parenteral treatment, with the others receiving oral chloroquine. Similarly, the one patient with vivax malaria, who also met severity criteria on admission, was treated only with oral chloroquine. In contrast, six of seven patients with severe falciparum malaria received parenteral treatment within two hours of diagnosis (p = 0.07 for fatal *P. falciparum vs* non-falciparum; Yates corrected Chi^2^). These findings suggest a lack of recognition of the potential of the non-falciparum malarias to cause severe disease and the need for treatment with intravenous artesunate for all severe malaria patients.

This study had several limitations, the major one being its retrospective design resulting in unavoidably incomplete laboratory and clinical data. Blood culture results were not available for two patients, and for these patients bacterial sepsis cannot be excluded as contributing to poor outcome. Lack of accurate parasite density counts also made assessment of parasite burden difficult. Post-mortem examination was not performed for any patient, preventing detailed description of the pathogenic mechanisms of severe malaria and death.

## Conclusion

*Plasmodium knowlesi* is a common cause of malaria in Sabah, and in this series was responsible, either directly or through bacteraemia, for almost half of malaria deaths throughout the state. These results highlight the importance of accurate reporting of *P. knowlesi* to improve recognition and management of this species. Clinicians need to be better informed of the potential for severe and fatal malaria from non-falciparum species, especially *P. knowlesi,* and the need to treat all severe malaria with immediate intravenous artesunate regardless of species.

## Competing interests

The authors declare that they have no competing interests.

## Authors’ contributions

GR performed the case note review with assistance from TW and BB. TW and JM facilitated the case review. GR, BB, NA and TY wrote the paper. All authors read and approved the final manuscript.
